# Role of ANT2 in mitochondrial function and cancer cell survival: a target for therapeutic intervention

**DOI:** 10.1038/s41420-025-02510-z

**Published:** 2025-05-08

**Authors:** Klara Bohacova, Zuzana Nahacka, Jana Dudova, Jaromira Kovarova, Jakub Rohlena, Michaela Rennerova, Barbora Judita Kasperova, Jan Stursa, Lukas Werner, Martin Haluzik, Jiri Neuzil, Sona Stemberkova Hubackova

**Affiliations:** 1 Institute of Biotechnology, Czech Academy of Sciences, Prague-West, Czechia; 2 Faculty of Science, Charles University, Prague, Czechia; 3 Centre for Experimental Medicine, Institute for Clinical and Experimental Medicine, Prague, Czechia; 4 Diabetes Centre, Institute for Clinical and Experimental Medicine, Prague, Czechia; 5 Department of Biochemistry and Microbiology, University of Chemistry and Technology, Prague, Czechia; 6 First Faculty of Medicine, Charles University in Prague, Prague, Czechia; 7 School of Pharmacy and Medical Science, Griffith University, Southport, QLD Australia

**Keywords:** Targeted therapies, Cancer therapy

Dear Editor,

Mitochondria play an indispensable role in cancer cell survival not only due to their role in energy production and metabolic regulation, but also for the regulation of de novo pyrimidine biosynthesis, which is critical for cancer cell replication and growth. By efficiently generating ATP by oxidative phosphorylation (OXPHOS), mitochondria meet the high energy demand of rapidly proliferating cancer cells. Disruption in OXPHOS however impairs not only ATP production, but also ubiquinone regeneration, which prevents dihydroorotate dehydrogenase activity and de novo pyrimidine production [1]. Additionally, they regulate apoptosis, allowing cancer cells to evade programmed cell death. Mitochondria also maintain reactive oxygen species (ROS) levels, balancing signaling and oxidative stress to support cellular adaptation and resilience. These features are critical for sustaining cancer cell growth, making mitochondria essential components in their survival and in the progression of malignancies.

We previously reported the importance of mitochondrial respiration in tumor formation and progression. It was shown that cancer cells without mitochondrial DNA (mtDNA; ρ^0^ cells) form tumors after considerable delay and only upon acquisition of functional mitochondria from the donor, resulting in respiration recovery [1]. Moreover, increased tumorigenesis corresponded not only to restored mitochondrial respiratory complexes and OXPHOS, but also to restored expression of polymerase γ (POLG) [1]. POLG is essential for replication and repair of mtDNA. It ensures the integrity and proper function of the mitochondrial genome, required for OXPHOS maintenance and energy generation [2]. Functional POLG plays a key role in tumor growth since POLG deficiency in cancer cells prevents tumor formation (Suppl. Fig. 1A, B). The lack of POLG abrogates respiration (Suppl. Fig. 1C) and mitochondrial inner membrane potential (Suppl. Fig. 1D), which results in changes in mitochondrial morphology (Suppl. Fig. 1E) similar to those observed in ρ^0^ tumor cells (Suppl. Fig. 1C-E). Despite promising results from preclinical studies [3], current POLG inhibitors do not specifically target tumor cells, which increases the risk of side effects on proliferating cells, leading to the search for new therapeutic approaches targeting mitochondrial metabolism.

Mitochondrial dysfunction resulting from POLG defects can activate multiple cellular stress responses, ultimately leading to decreased transcription of adenine nucleotide translocator 2 (ANT2) (Suppl. Fig. 1F, G) as a strategy to cope with impaired mitochondrial function [4]. Among the number of proteins that orchestrate mitochondrial function, the ANT family with its predominant isoforms ANT1 and ANT2 stands out for its pivotal role in cellular ATP/ADP management. The primary function of ANTs is to facilitate the exchange of ADP and ATP across the mitochondrial inner membrane, a process that is crucial for maintaining the energy balance within the cell. ANT2 has garnered significant attention in the context of cancer biology due to its multifaceted role in mitochondria of cancer cells. Cancer cells, known for their high metabolic activity, depend on efficient ATP production and distribution to support their accelerated growth and division. Unlike ANT1, ANT2 can transport ATP formed by glycolysis into mitochondria to generate the inner membrane potential [5]. ANT2 is therefore often overexpressed in cancer cells that rely on glycolysis [6], allowing them to maintain mitochondrial function in the state of reduced OXPHOS and to evade cell death. Indeed, inhibition or depletion of ANT2 increases cancer cell sensitivity to apoptotic stimuli [7], pointing to its potential as a target for cancer therapy. However, tumor cells exhibit metabolic plasticity, allowing them to evade metabolism targeting. Thus, simultaneous inhibition of ANT2 and mitochondrial metabolism, similar to POLG inhibition, may be an effective anti-cancer therapy, disrupting metabolic adaptations that support tumor growth.

Pancreatic ductal adenocarcinoma (PDAC), the most common type of pancreatic cancer accounting for about 80% of all cases, is an aggressive disease with approximately 500,000 newly diagnosed patients each year worldwide. Current therapy options, including surgery and chemotherapy, are limited, with an overall median survival of 14 months [8], highlighting the need for new therapeutic strategies. As an important regulator of cancer cell growth and survival ANT2 represents a valuable target for PDAC therapy. To assess feasibility of ANT2 targeting in PDAC and its potential synergy with OXPHOS disruption, we used carboxyatractyloside (CATR, 5 μM), an inhibitor of ANT channels [9]. CATR treatment decreased proliferation in PDAC cells (Fig. 1A for PaTu8902 cells, Suppl. Fig. 2A for MiaPaCa, AsPC1 and KPC-1 cells), which was associated with an increased level of ROS (Fig. 1B for PaTu8902 cells, Suppl. Fig. 2B for MiaPaCa, AsPC1 and KPC-1 cells). Mitochondrial fission was detected by confocal (Fig. 1C for PaTu8902 cells, Suppl. Fig. 2C for MiaPaCa, AsPC1 and KPC-1 cells; Suppl. Fig. 2D represents statistical evaluation of structural changes) and electron (Fig. 1D for PaTu8902 cells) microscopy. However, despite morphological changes, the structure of mitochondrial cristae remained unchanged (Fig. 1D), which is consistent with the limited impact of CATR treatment on the mitochondrial inner membrane potential (Fig. 1E for PaTu8902 cells, Suppl. Fig. 2E for MiaPaCa, AsPC1 and KPC-1 cells). Due to the high in vivo toxicity of CATR (summarized in [10]), mice with PDAC tumors were treated with Suramin, a drug against human sleeping sickness caused by trypanosomes which also inhibits ANT channel (besides interfering with ATP-binding and purinergic signaling) [11]. We observed that intraperitoneal administration of Suramin decreased the growth of PDAC tumors (Fig. 1F). To assess the specific role of ANT2 in this effect, we prepared PaTu8902, MiaPaCa and AsPC1 cells with doxycycline-inducible downregulation of ANT2 (shANT2) (Fig. 1G for PaTu8902 cells, Suppl. Fig. 2F for MiaPaCa and AsPC1 cells). Downregulation of ANT2 resulted in decreased proliferation of PDAC cells in vitro compared to their wild-type counterparts (Fig. 1H for PaTu8902 cells, Suppl. Fig. 2G for MiaPaCa and AsPC1 cells) and delayed tumor formation (Fig. 1I) as well as slower tumor growth kinetics in vivo (Fig. 1J-K). No effect on animal weight reduction indicating health problems was observed (Fig. 1L).

**Fig. 1:**
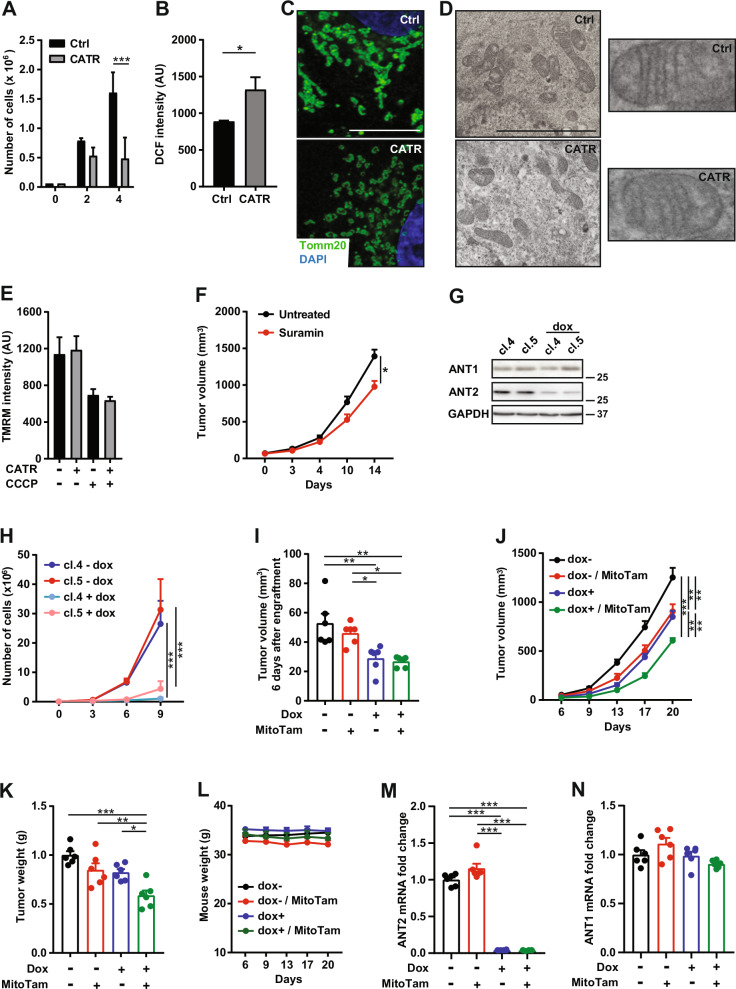
Simultaneous inhibition of ANT2 and OXPHOS reduces tumorigenesis in PDAC. **A** PaTu8902 cells were treated with carboxyatractiloside (CATR; 5μM) for 2 and 4 days. Proliferation was evaluated by Vi-CELL Series Cell Viability Analyzer. PaTu8902 cells were treated with CATR (5μM) for 96 h. **B** Reactive oxygen species were detected by 2´7´-dichlorofluorescein (DCF; 10 μM, 15 min) and analyzed by FACS. Mitochondrial morphology was documented by (**C**) confocal microscope following Tomm20 immunofluorescent staining, with DAPI denoting cell nuclei, and (**D**) by electron microscopy. The scale bar indicates 10 μm (**C**) and 0.5 μm (**D**). **E** Mitochondrial membrane potential (ΔΨ_m,i_) was detected by the fluorescent dye tetramethylrhodamine methyl ester (TMRM; 50 nM, 15 min) and analyzed by FACS. Carbonyl cyanide *m*-chlorophenyl hydrazine (CCCP; 20 μM) was added 5 min before TMRM to see specific suppression of ΔΨ_m,i_. **F** PaTu8902 cells (10^6^ in 100 μL of PBS) were grafted subcutaneously into immunodeficient NOD scid gamma (NSG) mice. When tumors reached approximatelly 50 mm^3^, mice (n = 6) were treated three times per week by intraperitoneal administration of Suramin (10 mg/kg) dissolved in physiological solution or the vehicle. **G** PaTu8902 cells transfected with shANT2 (clones 4 and 5) were exposed to doxycycline (1 μg/mL) for 72 h, and the levels of ANT1 and ANT2 were analyzed by immunoblot. GAPDH was used as a loading control. **H** Proliferation was evaluated by hemocytometer at the times indicated (0, 3, 6 and 9 days after the addition of doxycycline - 1 μg/mL). PaTu8902 cells (10^6^ in 100 μL of PBS) transfected with shANT2 cells (mix of clones 4 and 5) pre-treated with doxycycline (1 µg/mL) for 72 h were grafted subcutaneously into NSG mice (n = 6). Mice were subsequently administered doxycycline in water (0.4 mg/mL) throughout the experiment. shANT2 PaTu8902 cells without doxycycline pre-treatment/ administration were used as a control. Six days after engraftment, mice were treated twice per week by intraperitoneal administration of MitoTam (MT; 4 mg/kg) dissolved in 4% EtOH in corn oil or the vehicle (corn oil; CO). **I** Tumor volume on day 6 after engraftment of tumor cells. **J** Tumor volume was determined using a caliper at the times indicated. **K** Tumor weight at the end of the experiment. **L** Weight of animals during the experiment. Tumor tissue was assessed for expression of **M** *ANT2* and **N** *ANT1* using RT-qPCR. *β-Actin* was used as a reference gene.

Despite ANT2 downregulation, ANT1 expression remained unchanged (Fig. 1M, N), excluding secondary ANT1-related effects. ANT2 helps maintain mitochondrial integrity in the face of oxidative stress, promoting survival. Its upregulation in cancer cells contributes to resistance to therapies that rely on increase in oxidative stress. ANT1, in contrast, is less effective at protecting against oxidative stress and is more likely to trigger cell death in response to high ROS levels. Combined downregulation of ANT2 and inhibition of OXPHOS using MitoTam, a mitochondrially targeted anti-cancer agent with a dual effect on mitochondrial membrane depolarization and inhibition of respiration [12], which demonstrated strong safety profile during Phase 1/1b clinical trial [13], resulted in synthetic reduction of tumor growth compared to inhibition of OXPHOS or ANT2 alone (Fig. 1I-L).

In summary, we show that ANT2 is an important component of the mitochondrial machinery in PDAC cancer cells, which could be targeted pharmacologically. This warrants further investigation into the biological role of ANT2 in PDAC. Furthermore, our findings of potentiated effect between ANT2 disruption and OXPHOS inhibition by MitoTam suggest that this combination offers a potentially novel approach for therapeutic targeting of PDAC tumors (see Suppl. Fig. 3 for scheme). As current ANT inhibitors suffer from low specificity/efficacy (e.g. Suramin) [14] or high toxicity (CATR), our results provide a rationale for developing new, more specific ANT2 inhibitors for use in PDAC.

## Supplementary information

Supplemental material

Original Data

## Data Availability

All data are available in the main text or the supplementary materials.
