# Supplementary material for: Role of ANT2 in mitochondrial function and cancer cell survival: a target for therapeutic intervention
Source: Cell Death Discov. 2025 May 8;11:225. doi: 10.1038/s41420-025-02510-z (PMC12059193; doi:10.1038/s41420-025-02510-z)

MATERIAL AND METHODS

Material

Carboxyatractyloside (PHL84196) was ordered from Sigma (Rahway, NJ, USA). Suramin (CAS 129-46-4) was ordered from Henan Tainfu Chemicals (Zhengzhou, China).

Cell lines

Human pancreatic adenocarcinoma cells PaTu8902, MiaPaCa and AsPC1 were purchased from ATCC (Manassas, VA, USA). PaTu8902 and MiaPaCa cells were cultured in DMEM medium supplemented with 10% foetal bovine serum (FBS) (ThermoFisher Scientific, Waltham, MA, USA), 1 g/L glucose (Sigma) and 100 U/mL penicillin and 100 μg/mL streptomycin sulfate (P/S) (Sigma). AsPC1 cells were grown in DMEM medium supplemented with 10% FBS (ThermoFisher Scientific), 4.5 g/L glucose (Sigma), and P/S (Sigma). KPC-1 cells were obtained from dr. Kalyaranaman, Medical College of Wisconsin, USA. Cells were maintained in the DMEM medium supplemented with 10% FBS (ThermoFisher Scientific), 4.5 g/L glucose (Sigma), and P/S (Sigma).

4T1 mouse metastatic breast cancer cells were purchased from the ATCC (Manassas, VA, USA) and maintained in the RPMI medium with 10% FBS and P/S.

4T1 ρ0 cells were prepared as described (1). Cells were cultivated in the RPMI medium with 10% FBS and P/S in the presence of 1 mM pyruvate and 50 mg/mL uridine. Prior experiment, cells were cultivated for 72 h in the absence of pyruvate and uridine.

The cells were kept at 37 °C under 5 % CO_2 in a humidified atmosphere.

4T1 POLG knockout cells

Custom-made CRISPR/Cas9 plasmid system trageting sequence of mouse POLG (Sigma, 03091517MN) was used for lipofectamine 3000 (Thermo Fisher Scientific, L3000008) transfection of 4T1 cells. Cells were incubated with Cas9 system for 48 h and then sorted according to expression of reporter gene cassette mCherry. The knockout of Polγ was verified by protein expression using immunoblotting. POLG KO cells were cultivated in the RPMI medium with 10% FCS and antibiotics in the presence of 1 mM pyruvate and 50 mg/mL uridine. Prior experiment, cells were cultivated for 72h in the absence of pyruvate and uridine

Preparation of shANT2 cells

Cells with downregulated ANT2 were prepared by using the inducible pLKO tet-on shRNA system as following. Unless stated otherwise, all enzymes were purchased from Thermo Fisher Scientific and used according to the manufacturer´s instructions. Oligos of shANT2 (Sigma, TRCN0000230805: CCGCCTACTTCGGTATCTATGCTCGAGCATAGATACCGAAGTAGGCGGTTTTT, TRCN0000230804: GTGTCTGTGCAGGGTATTATCCTCGAGGATAATACCCTGCACAGACACTTTTT) were annealed (37 °C/30 min, 95 °C/5 min, 25 °C ramp down 5%/min) and ligated into Age1/EcoRI digested (FD1464 and FD0274 respectively) pLKO tet-on empty vector (Addgene # 21915) using T4 DNA ligase (15224017). The prepared constructs were transformed into Stbl3 bacterial strain (C737303), verfied using colony PCR (DreamTaq Green PCR Master Mix 2X, K1081) and products of end-point PCR were analyzed by DNA electrophoresis (2% agarose, 60 V/90 min) (AG02; Nippon Genetics, Duren, Germany). Plasmid DNA was isolated by NucleoBond Xtra Midi kit (740410; Macherey-Nagel, Hoerdt Cedex, France) following the manufacturer´s instructions. The constructs were verified by Sanger sequencing (Eurofins Genomics, Ebersberg, Germany; forward: H1 promoter GCTATGTGTTCTGGGAAATC, reverse: IRES CACACCGGCCTTATTCCAAG). Cells were then generated by transduction with lentiviral particles containing prepared constructs as published (2) and selected using puromycin antibiotics (2 μg/ml; ant-pr-1, Thermo Fisher Scientific). The expression of shRNA was activated by cultivating cells in the presence of doxycycline (1 μg/ml/48hrs; D1822, Sigma).

Evaluation of mitochondrial membrane potential and reactive oxygen species

Cells (6×10^4 on 12-well plate) were treated with CATR (5μM) 24h after seeding for 48h. To assess $\Delta\Psi_{m,i}$ and ROS production, cells were treated with potentiometric fluorescent dye tetramethylrhodamine methyl ester (TMRM; 50 nM) and 2´7´-dichlorofluorescein (DCF; 10 μM), respectively, for 15 min prior to analysis by flow cytometry (BD FACSMelody cell sorter, San Jose, CA, USA). Carbonyl cyanide *m*-chlorophenyl hydrazine (CCCP; 20 μM) was added 5 min before TMRM to see specific suppression of $\Delta\Psi_{m,i}$. Cells without added TMRM or DCF probes were used as a control of nonspecific signal.

Evaluation of respiration

The high-resolution Oxygraph-2k respirometer (Oroboros Instruments, Innsbruck, Austria) was used to assess routine respiration. Cells were trypsinized, washed with PBS, re-suspended at $2x10^6$ cells per mL in the Mir05 medium (0.5 mM EGTA, 3 mM $MgCl_2$, 60 mM K-lactobionate, 20 mM taurine, 10 mM KH_2PO_4, 110 mM sucrose, 1 g/L essentially fatty acid-free bovine serum albumin, 20 mM HEPES, pH 7.1 at 30 °C) and transferred to the chamber of the Oxygraph-2k instrument. Respiration evaluation was performed at 37 °C. After signal stabilization, the chamber was closed, and the oxygen consumption was measured, showing the level of basal respiration. To measure complex I (CI) and complex II (CII) dependent respiration, malonate (10 µg/ml) and rotenone (2 µg/ml) were added to inhibit CII and CI, respectively. The level of CI and CII respiration was determined following the addition of glutamate/malate (5/5 µg/ml) and succinate c (10 µg/ml), respectively. Data were evaluated using DatLab5 software (Oroboros Instruments, Innsbruck, Austria).

Electrophoresis and western blotting

Cells were washed twice with PBS, harvested into Laemmli SDS sample lysis buffer (2% SDS, 50 mM Tris-Cl, 10% glycerol in double-distilled H_2O) and sonicated (2x10 s at 1 micron amplitude with 10 s cooling interval) using the Soniprep 150 instrument (MSE, London, UK). Protein concentration was estimated using the BCA method (Thermo Fisher Scientific). Cell lysates were supplemented with 100 mM DTT (Sigma) and 0.01% bromophenol blue (Sigma) before separation by SDS-PAGE. The same amount of protein (50-70 µg) was loaded into each well. The protein was transferred onto a nitrocellulose membrane using wet transfer and detected by specific antibodies combined with horseradish peroxidase-conjugated secondary antibodies (goat anti-rabbit). Peroxidase activity was detected using the ECL Western Blotting Substrate or the SuperSignal West Femto Extended Duration Substrate (Thermo Fisher Scientific). anti-ANT1 (ab102032) was ordered from Abcam (Cambridge, UK) and anti-ANT2 (#14671) and anti POLG (#13609) were ordered from Cell Signaling (Danvers, MA, USA). All antibodies were diluted 1:1,000 in 2.5% non-fat milk. HPR conjugated β-actin (PA1-183-HPR; Thermo Fisher Scientific) or GAPDH (#5174; Cell Signaling) were used as a loading control. IgG-HRP anti-rabbit (170-6515) secondary antibody produced in goat was purchased from BioRad Laboratories (Hercules, CA, USA). The secondary antibody was diluted 1:10,000 in 2.5% non-fat milk.

Indirect immunofluorescence

Cells (3×10^4 on 24-well plate) grown on glass coverslips were fixed with 4 % paraformaldehyde (PFA; VWR, Radnor, PA, USA) and permeabilized with 0.1 % Triton X-100 (Sigma) in two consecutive steps, each at room temperature (RT) for 15 min. After washing with PBS, cells were incubated in 10 % FBS (diluted in PBS) for 30 min to block unspecific signals. Cells were incubated for one hour at RT with diluted primary antibody anti-Tomm20 (EPR15581-54, Abcam), washed extensively with PBS/0.1 % Tween 20, and incubated for one hour at RT with secondary antibody goat anti-rabbit Alexa Fluor 488 (A11006, Thermo Fisher Scientific). Coverslips were mounted in Mowiol containing 4',6-diamidino-2-phenylindole (DAPI; Sigma) to stain nuclei, and the signal was detected using the Leica SP8 FLIM confocal microscope (Leica Microsystems, Wetzlar, Germany). Structural changes of mitochondria were evaluated using ImageJ with MiNa (Mitochondrial Network Analysis) plugin.

Quantitative real-time PCR (qRT-PCR)

Small pieces of tissue (1-2 mm^3) were placed into 500 μL of RNAzol (BioRad) and homogenized (3 x 40 s at 5,600 rpm) using the Precellys 24 homogenizer (Bertin Instruments, Montigny-le-Bretonneux, France). Cells (500 000) were lysed in 500 μL of RNAzol by repeated pipetting. Total RNA was isolated according to the manufacturer´s protocol. First strand cDNA was synthesized from 1 μg of total RNA with random hexamer primers using Revert Aid First strand cDNA Synthesis Kit (Thermo Fisher Scientific). RT-qPCR was performed using the CFX384 Touch Real-Time PCR Detection System (BioRad) with 5xHOT FIREPol Evagreen qPCR Supermix GreenE dye (Solis Biodyne, Tartu, Estonia). The relative quantity of cDNA was estimated by the ΔΔCT method, data were normalized to β-actin. The following primers were purchased from Sigma: ANT1: forward 5´- GCTGCCTACTTCGGAGTCTATG-3´, reverse 5´-TGCGACTGCCGTCACACTCTG-3´; ANT2: forward 5´-GCCGCCTACTTCGGTATCTATG-3´, reverse 5´-CAGCAGTGACAGTCTGTGCGAT-3´; β-actin: forward 5´-CCAACCGCGAGAAGATGA-3´, reverse 5´-CCAGAGGCGTACAGGGATAG-3´; mouse ANT2: forward 5´- ACACGGTTCGCCGTCGTATGAT-3´, reverse 5´-AAAGCCTTGCTCCCTTCATCGC-3´; mouse β-actin: forward 5´-CAT TGC TGA CAG GAT GCA GAA GG-3´, reverse 5´-TGC TGG AAG GTG GAC AGT GAG G-3´.

Cell growth and viability

Cells (6×10^4 on 12-well plate) were treated with CATR (5μM) 24h after seeding. shANT2 cells (3×10^4 on 24-well plate) were treated with doxycycline (1 μg/ml) immediately after seeding. Cell viability was assessed 48 and 96 hours after the addition of treatment by Vi-CELL Series Cell Viability Analyzer (Beckman Coulter) using trypan blue staining to differentiate viable and non-viable cells. Prior to measurement, the cells were trypsinized to ensure a uniform single-cell suspension.

Transmission electron microscopy (TEM)

TEM was performed according to a standard protocol. In brief, cells (3×10^4 on 24-well plate) were grown on cover-slips, fixed with 2.5% glutaraldehyde (Sigma) overnight, and post-fixed with 1% OsO4 (Sigma) made up in Sorensen's phosphate buffer (0.1 M, pH 7.2-7.4), dehydrated in acetone series, and embedded in Epon-Durcupan (Sigma). Ultrathin sections (~70-90 nm) were cut, contrasted with uranyl acetate (Ladd Research Industries, Williston, VT, USA), and examined in the JEM-1400 FLASH transmission electron microscope (Jeol) at 80 kV. Images were captured with 2kx2k FLASH CMOS camera.

Animal experiments

PaTu8902 cells (1 mil. in 100 μL of PBS) were grafted subcutaneously into immunodeficient NOD scid gamma (NSG) mice (males; 8-10 weeks old). When tumours reached approximately 50 mm^3, mice (n=6) were treated three times per week by intraperitoneal administration with Suramin (10 mg/kg) dissolved in physiological solution or the vehicle. At the end of the experiment, tumors were collected and frozen at -80°C until further analysis.

PaTu8902 cells (1 mil. in 100 μL of PBS) transfected with shANT2 cells (mix of clones 4 and 5) pre-treated with doxycycline (1 μg/mL) for 72 h were grafted subcutaneously into NSG mice (n=6; males, 10-12 weeks old). Mice were subsequently administered doxycyclin in water (0.4mg/mL) throughout the experiment. Water with doxycycline was changed every day. shANT2 PaTu8902 cells without doxycycline pre-treatment/administration were used as a control. 6 days after engraftment, mice were treated twice per week by intraperitoneal administration of MitoTam (4 mg/kg) dissolved in 4% EtOH in corn oil or the vehicle. At the end of the experiment, tumors were collected and frozen at -80°C until further analysis.

Parental and Polγ-/- 4T1 cells (1 mil. in 100 μL of PBS) were grafted subcutaneously into Balb-c mice (n=6; females, 10-12 weeks old). Tumor volume was measured by caliper.

Allocation of animals into groups were based on body weight and tumor volume at the D0 to achieve approximately equal values within groups. All mice were maintained at 22 °C and 12 h/12 h light/dark regimen with food and water provided *ad libitum*. These experiments were performed in agreement with the Animal Protection Law of the Czech Republic and were approved by the Ethics Committee of the Institute of Molecular Genetics, Prague.

Statistical analysis

Statistical analysis was performed, and graphs were drawn using GraphPad Prism software. One-way or Two-way ANOVA followed by Tukey's multiple comparisons test presented as mean ± SD for *in vitro* data and mean ± SEM for *in vivo* data was used. Assumptions of normality was verified using Shapiro-Wilk test. Statistical significance with * $p<0.05$, ** $p<0.01$, *** $p<0.005$ was considered as significant. In vitro experiments were measured in biological triplicates (each triplicate was measured in technical duplicate).

LEGENDS TO SUPPLEMENTARY FIGURES

Suppl. Figure 1

(A) Parental and POLG-/- 4T1 cells (1 mil. in 100 μL of PBS) were grafted subcutaneously into Balb-c mice (n=5). Two clones of POLG-/- 4T1 cells were tested (clone1 and clone 2). Tumor volume was measured by caliper. **(B)** The level of POLG in parental (P) and POLG-/- 4T1 cells (clones 1 and 2) was analyzed by immunoblot. β-actin was used as a loading control. **(C)** Routine respiration was determined using the Oxygraph. **(D)** Mitochondrial membrane potential ($\Delta\Psi_{m,i}$) in parental, POLG-/- and ρ0 4T1 cells was detected by tetramethylrhodamine methyl ester (TMRM; 50 nM, 15 min) and analyzed by FACS. Carbonyl cyanide *m*-chlorophenyl hydrazine (CCCP; 20 μM) was added 5 min before TMRM to see specific suppression of $\Delta\Psi_{m,i}$. **(E)** Mitochondrial morphology was documented by Tomm20 immunofluorescent staining, with DAPI denoting cell nuclei. The scale bar indicates 10 μm. **(F)** The level of ANT2 was analyzed by immunoblot. β-actin was used as a loading control. **(G)** Expression of *ANT2* was assessed using RT-qPCR. *β-Actin* was used as a reference gene.

Suppl. Figure 2

MiaPaCa, KPC-1 and AsPC1 cells were treated with carboxyatractiloside (CATR; 5μM) for 48 and 96 h. **(A)** Proliferation was evaluated by Vi-CELL Series Cell Viability Analyzer. MiaPaCa, KPC-1 and AsPC1 cells were treated with CATR (5μM) for 96 h. **(B)** Reactive oxygen species were detected by 2´7´-dichlorofluorescein (DCF; 10 μM, 15 min) and analyzed by FACS. (C) Mitochondrial morphology was documented by confocal microscope following Tomm20 immunofluorescent staining, with DAPI denoting cell nuclei. The scale bar indicates 10 μm. (D) Statistical evaluation of mitochondrial morphology was performed using ImageJ software with MiNa (Mitochondrial Network Analysis) plugin. **(E)** Mitochondrial membrane potential ($\Delta\Psi_{m,i}$) was detected by the fluorescent dye tetramethylrhodamine methyl ester (TMRM; 50 nM, 15 min) and analyzed by FACS. Carbonyl cyanide *m*-chlorophenyl hydrazine (CCCP; 20 μM) was added 5 min before TMRM to see specific suppression of $\Delta\Psi_{m,i}$. **(F)** MiaPaCa and AsPC1 cells transfected with shANT2 (clones 4 and 5) were exposed to doxycycline (1 μg/mL) for 48h, and the levels of ANT2 was analyzed by immunoblot. β-actin was used as a loading control. **(G)** Proliferation was evaluated by Vi-CELL Series Cell Viability Analyzer at the times indicated.

Suppl. Figure 3

ANT2, unlike ANT1, can transfer cytosolic ATP generated by glycolysis to the mitochondria and thereby maintain mitochondrial potential and mitochondrial integrity when OXPHOS is compromised, reducing the activation of cell death due to dysfunctional mitochondria. Therefore, simultaneous inhibition of OXPHOS by MitoTam and ANT2 translocator increases tumor cell death.

Reference:

1. Tan AS, Baty JW, Dong LF, Bezawork-Geleta A, Endaya B, Goodwin J, et al. Mitochondrial genome acquisition restores respiratory function and tumorigenic potential of cancer cells without mitochondrial DNA. Cell Metab. 2015;21(1):81-94.

2. Nahacka Z, Svadlenka J, Peterka M, Ksandrova M, Benesova S, Neuzil J, et al. TRAIL induces apoptosis but not necroptosis in colorectal and pancreatic cancer cells preferentially via the TRAIL-R2/DR5 receptor. Biochim Biophys Acta Mol Cell Res. 2018;1865(3):522-31.

Supplemetary Figure 1

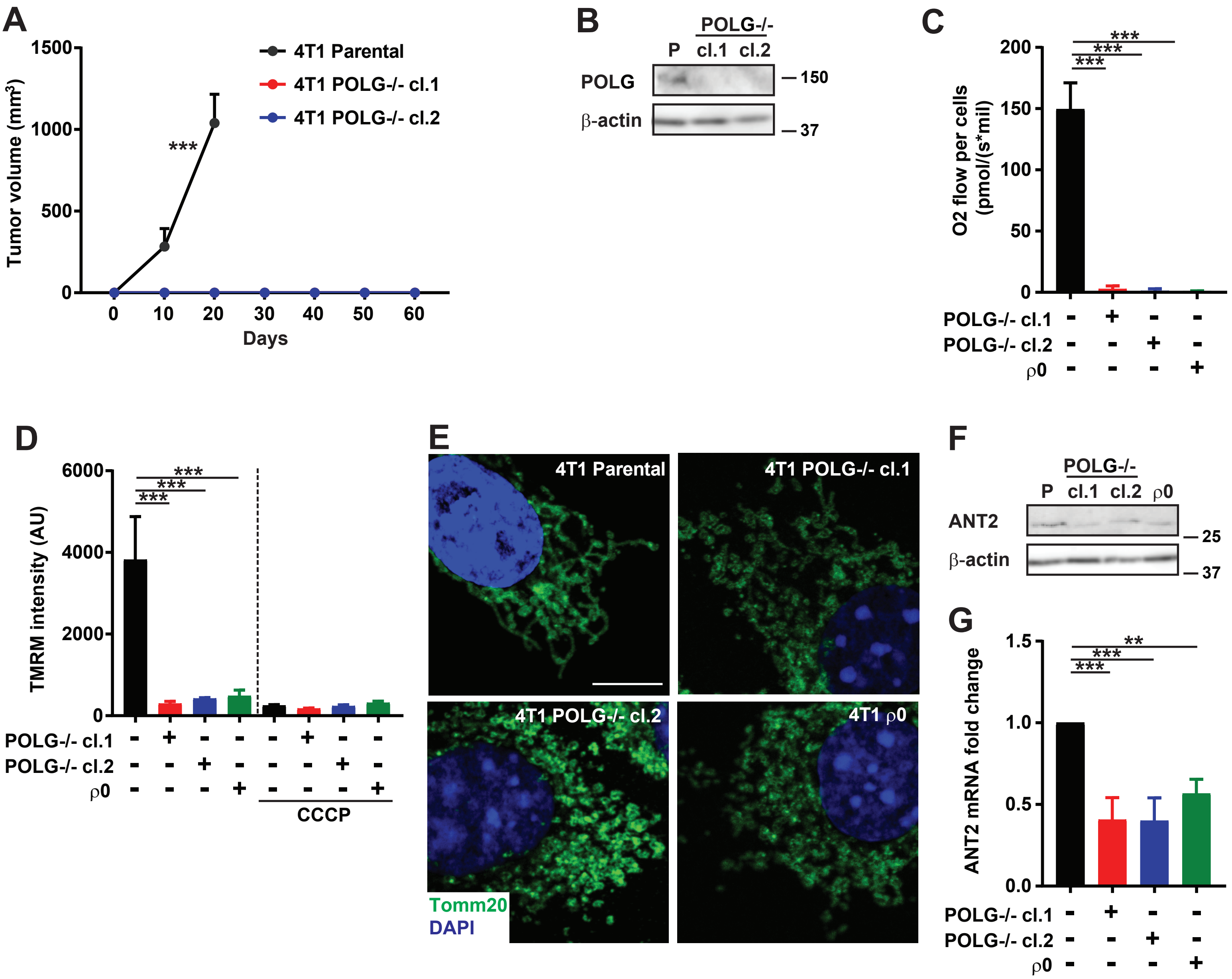

Supplemetary Figure 2

A

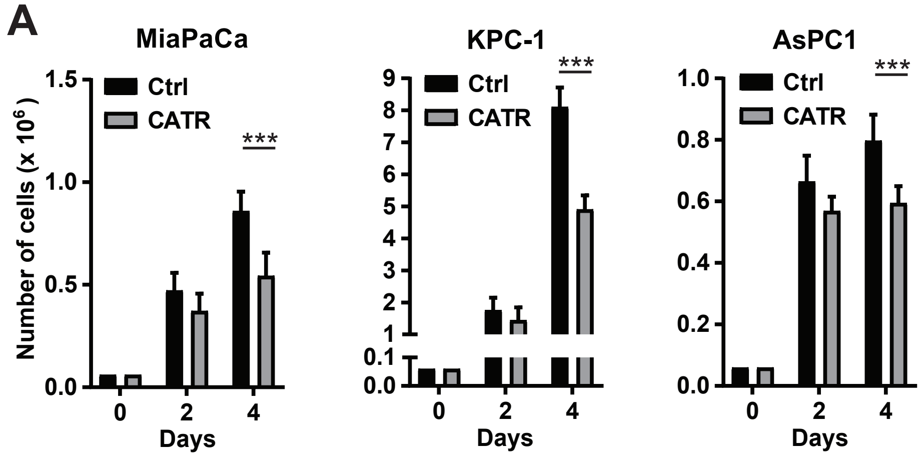

B

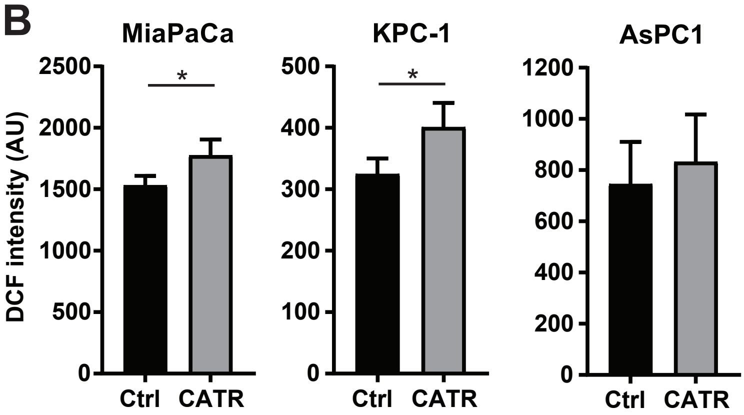

D

	PaTu		MiaPaCa	
	Ctrl	CATR	Ctrl	CATR
Individuals (n)	49,7±24,6	64,1±29,3***	27,7±15,2	22,4±17,2
Networks (n)	4,3±2,3	7,6±4,3***	4,4±2,4	3,17±2,7*
Mean Network Size (Branches)	72,3±42,9	43,6±22,4***	39,4±22,8	26,4±22,3**
	KPC-1		AsPC1	
	Ctrl	CATR	Ctrl	CATR
Individuals (n)	37,4±15,1	40,2±13,6	13,3±5,3	24,7±12,9***
Networks (n)	4,9±2,7	6,3±2,9**	2,6±1,1	5,2±2,1***
Mean Network Size (Branches)	38,7±30,4	33,7±37,5	30,1±18,5	16,5±8,1***

C

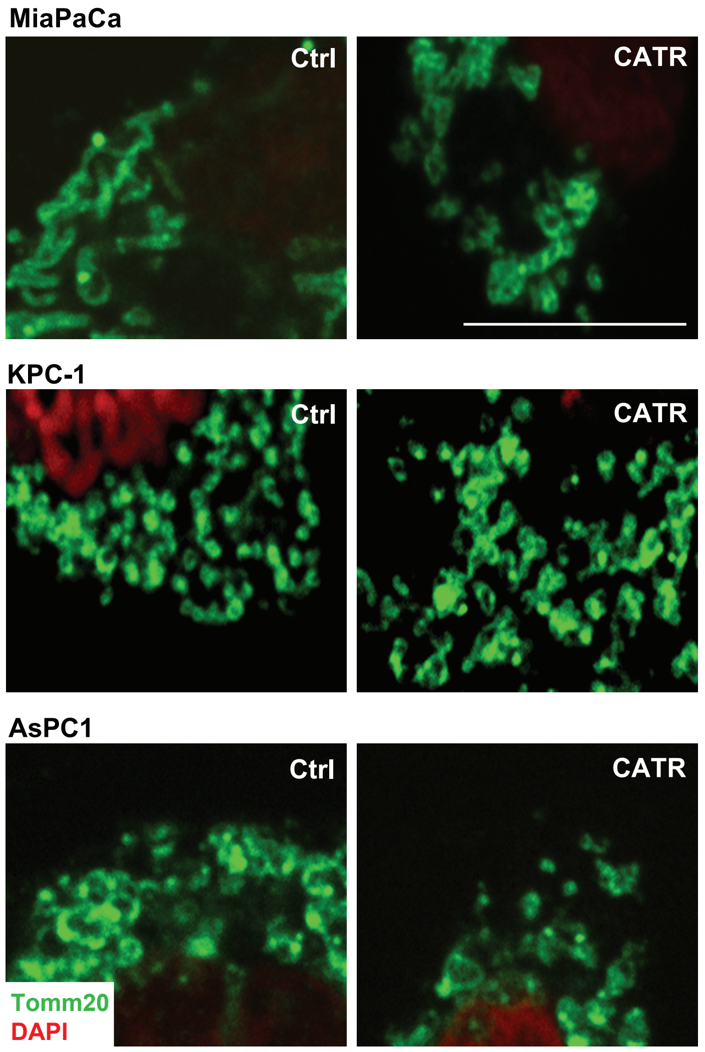

E

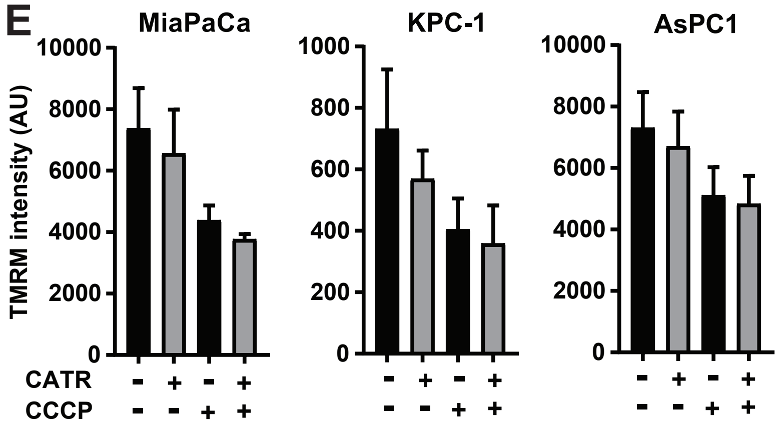

F

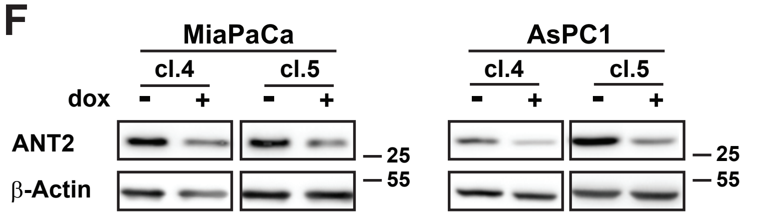

G

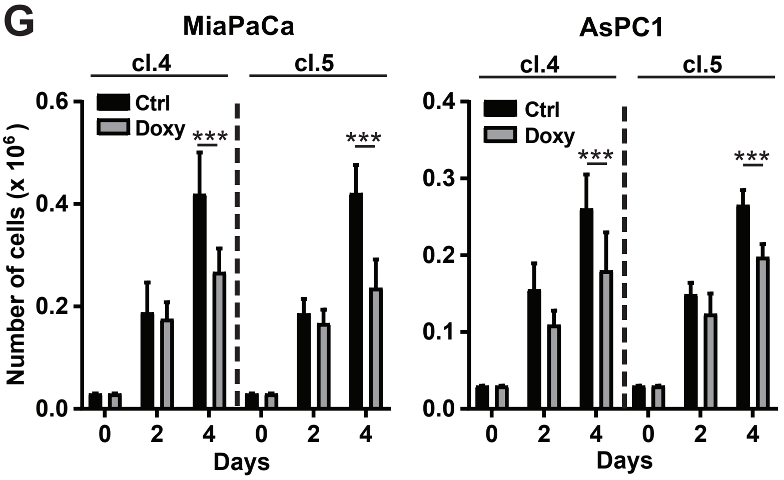

Supplemetary Figure 3

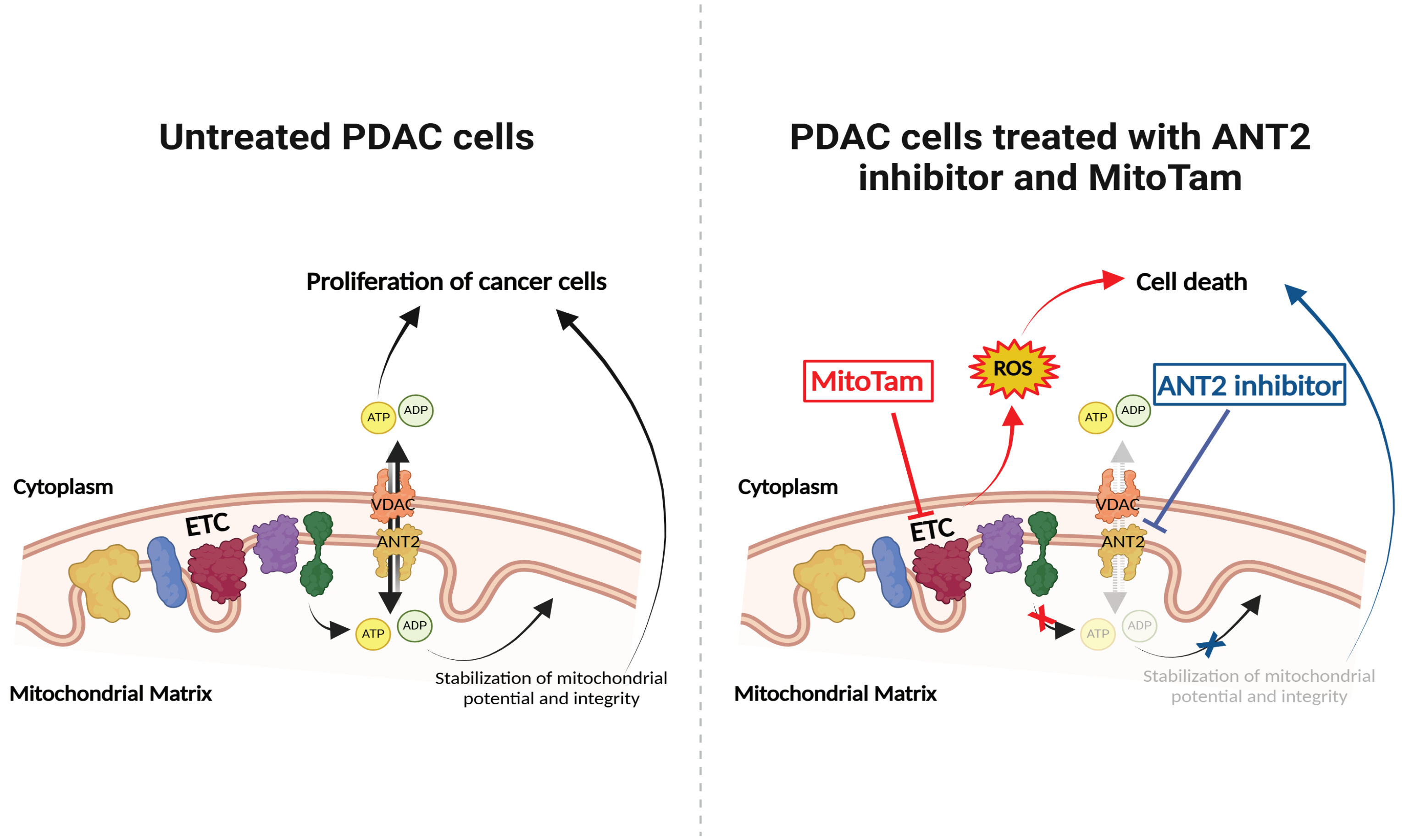

Uncropped immunoblots:

Supplementary Figure 1B – Actin

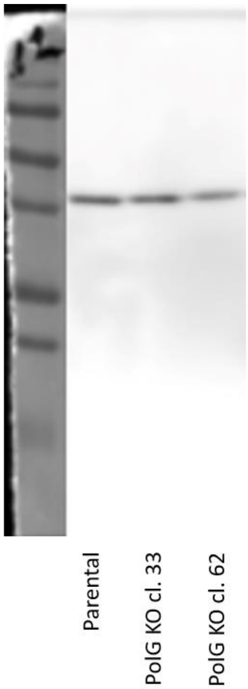

Supplementary Figure 1B – POLG

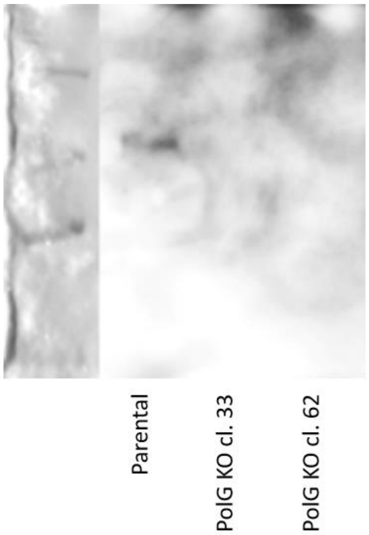

Supplementary Figure 1F – Actin

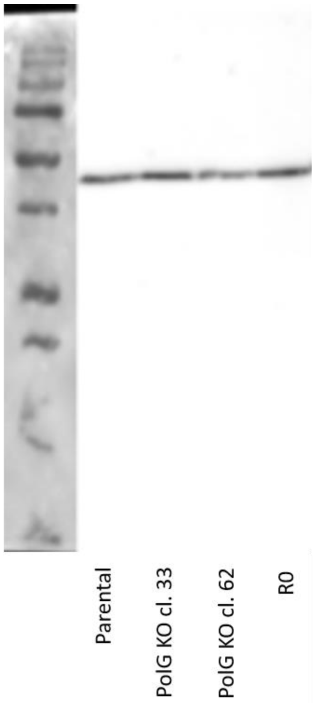

Supplementary Figure 1F – ANT2

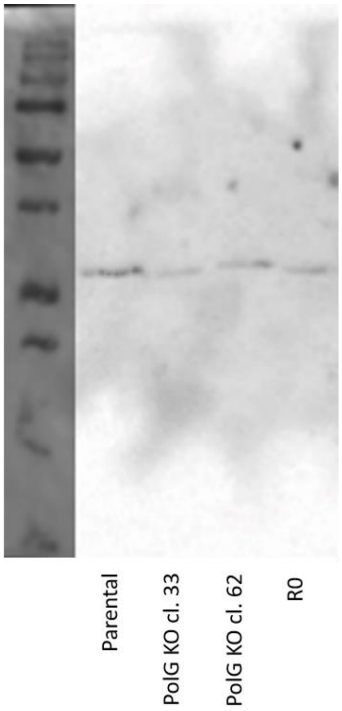

Supplementary Figure 2F (AsPC1) – Actin

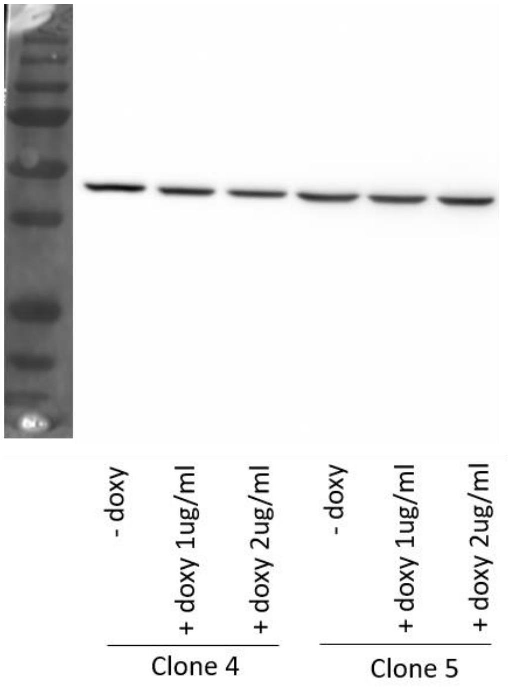

Supplementary Figure 2F (AsPC1) – ANT2

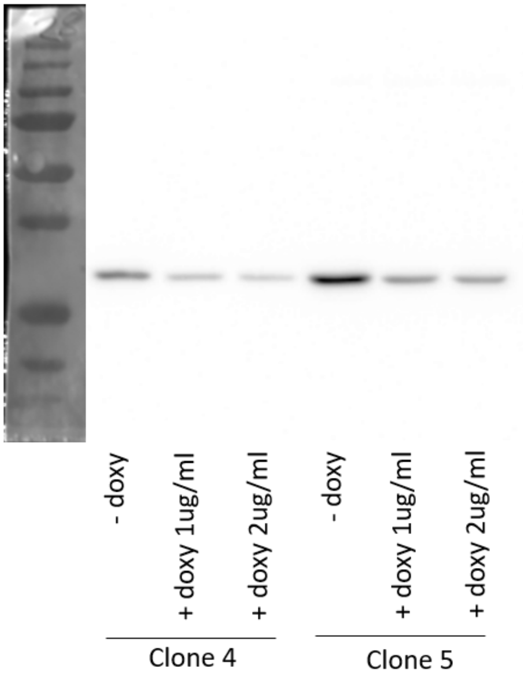

Supplementary Figure 2F (MiaPaCa) – Actin

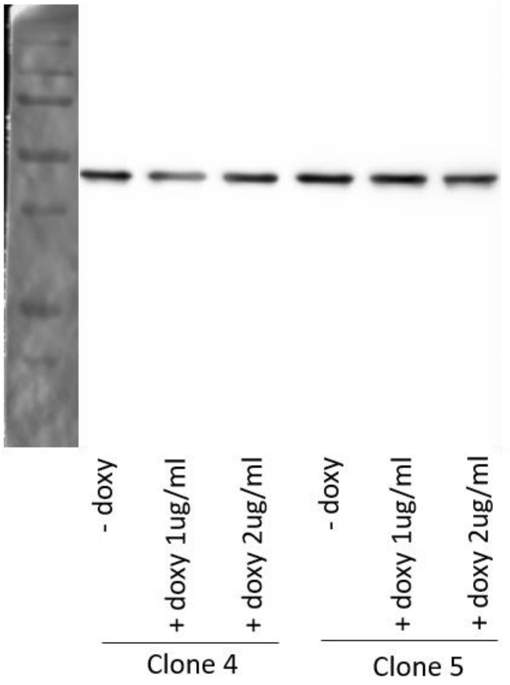

Supplementary Figure 2F (MiaPaCa) – ANT2

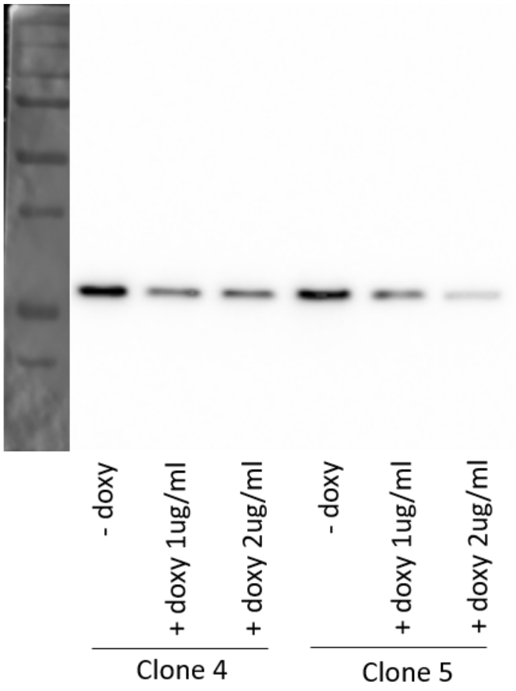

Supplement: Supplementary file 1 — Supplemental material [file 41420_2025_2510_MOESM1_ESM.pdf]
