# Supplementary figures and images for: Role of ANT2 in mitochondrial function and cancer cell survival: a target for therapeutic intervention
Source: Cell Death Discov. 2025 May 8;11:225. doi: 10.1038/s41420-025-02510-z (PMC12059193; doi:10.1038/s41420-025-02510-z)

Uncropped immunoblots:

Figure 1G – ANT2 Figure 1G – GAPDH


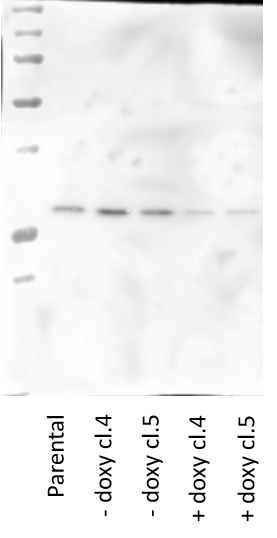

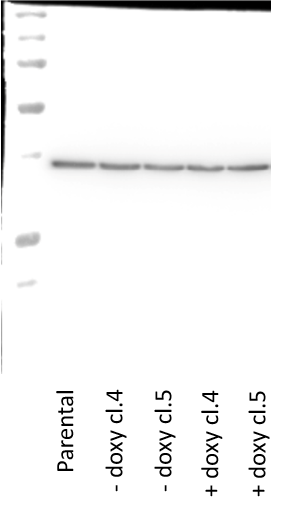


Figure 1G – ANT1


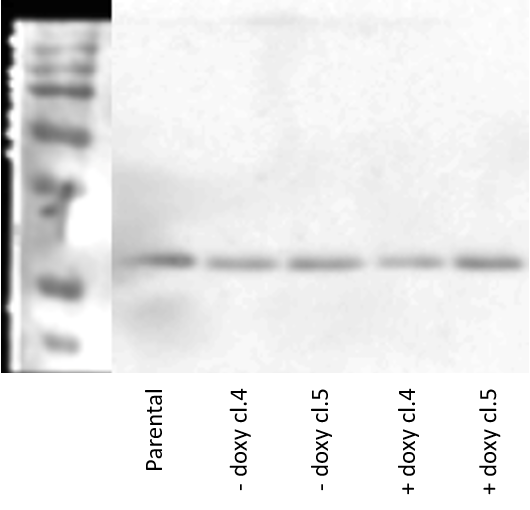

Supplement: Supplementary file 2 — Original Data [file 41420_2025_2510_MOESM2_ESM.docx]
